# Heart-to-heart: infant heart rate at 3 months is linked to infant-directed speech, mother–infant interaction, and later language outcomes

**DOI:** 10.3389/fnhum.2024.1380075

**Published:** 2024-05-02

**Authors:** Yaara Endevelt-Shapira, Alexis N. Bosseler, T. Christina Zhao, Julia C. Mizrahi, Andrew N. Meltzoff, Patricia K. Kuhl

**Affiliations:** ^1^Institute for Learning & Brain Sciences, University of Washington, Seattle, WA, United States; ^2^Department of Speech and Hearing Sciences, University of Washington, Seattle, WA, United States; ^3^Department of Psychology, University of Washington, Seattle, WA, United States

**Keywords:** heart rate, social interaction, language development, infant-directed speech, conversational-turns

## Abstract

**Introduction:**

Previous studies underscore the importance of speech input, particularly infant-directed speech (IDS) during one-on-one (1:1) parent–infant interaction, for child language development. We hypothesize that infants’ attention to speech input, specifically IDS, supports language acquisition. In infants, attention and orienting responses are associated with heart rate deceleration. We examined whether individual differences in infants’ heart rate measured during 1:1 mother–infant interaction is related to speech input and later language development scores in a longitudinal study.

**Methods:**

Using a sample of 31 3-month-olds, we assessed infant heart rate during mother–infant face-to-face interaction in a laboratory setting. Multiple measures of speech input were gathered at 3 months of age during naturally occurring interactions at home using the Language ENvironment Analysis (LENA) system. Language outcome measures were assessed in the same children at 30 months of age using the MacArthur-Bates Communicative Development Inventory (CDI).

**Results:**

Two novel findings emerged. First, we found that higher maternal IDS in a 1:1 context at home, as well as more mother–infant conversational turns at home, are associated with a lower heart rate measured during mother–infant social interaction in the laboratory. Second, we found significant associations between infant heart rate during mother–infant interaction in the laboratory at 3 months and prospective language development (CDI scores) at 30 months of age.

**Discussion:**

Considering the current results in conjunction with other converging theoretical and neuroscientific data, we argue that high IDS input in the context of 1:1 social interaction increases infants’ attention to speech and that infants’ attention to speech in early development fosters their prospective language growth.

## 1 Introduction

Language development can be modulated by different social and behavioral factors including socioeconomic status (Hart and Risley, 1995; Fernald et al., 2013) and early social interaction (Carpenter et al., 1998; Brooks and Meltzoff, 2005; Lytle and Kuhl, 2017; Rowe and Snow, 2020; Rowe and Weisleder, 2020; Bosseler et al., 2024). The course of early language learning has been shown to be influenced by the quality of early parent–child social interactions (Tamis-LeMonda et al., 2014; Madigan et al., 2019). For example, previous studies underscore the importance of infant-directed speech (IDS) and parent–child conversational turns (CTs) for child language development. IDS is a speech style that is characterized by a higher pitch, slower tempo, and exaggerated intonation contours when compared to standard adult-directed speech (Fernald and Simon, 1984). Infants exposed to more IDS in the home environment show larger expressive vocabulary later in development, a pattern that has been established in Spanish-learning infants (Weisleder and Fernald, 2013), English-learning infants (Ramírez-Esparza et al., 2014, 2017a) and in Spanish–English bilingual families (Ramírez-Esparza et al., 2017b). Similarly, the amount of adult–child CTs is positively associated with language development measurements (Zimmerman et al., 2009; Gilkerson et al., 2018; Ferjan Ramírez et al., 2020; Donnelly and Kidd, 2021; Huber et al., 2023; Nguyen et al., 2023).

Although these social and behavioral factors have been well studied in relation to language development, infant physiological state has received little to no attention as a potential factor in language development. In the current study, we investigated whether infant heart rate (HR) is related to speech input in the home environment and to prospective language development in a longitudinal study. To date, the few studies that have explored the association between infant HR and complex auditory input report a pattern of decrease in infant HR following exposure to IDS (Santesso et al., 2007; Curtindale et al., 2019; Lin et al., 2023) and musical stimuli (Schmidt et al., 2003). Heart rate has long been used as a physiological marker of attention (Sokolov, 1963; Graham and Jackson, 1970; Porges, 1992; Courage et al., 2006), and research indicates that attention is associated with early language learning (Çetinçelik et al., 2021 for review). Based on this previous work, we sought to examine whether infant HR during social interaction is related to: (a) the quality of speech input to the child at home and (b) children’s later language development in a longitudinal study design.

According to the “social gating” hypothesis (Kuhl, 2007), language learning is enhanced in social environments because it provides *information* that assists learning and *motivation* that increases learning. Infants’ increased attention to adults’ social-communicative intentions provides information that assists learning, and the mere presence of other humans in learning situations increases motivation, which enhances language learning (Kuhl et al., 2003; Lytle et al., 2018). These social factors play a role in the heightened saliency of linguistic input.

The role of attention in language learning was previously examined using infants’ eye-gaze behavior (Çetinçelik et al., 2021). For example, a laboratory-based study revealed that infants’ social and attentional behaviors during language exposure sessions, such as infants’ gaze shifts between a newly shown object and the tutor, were associated with neural measures of infants’ learning of new sounds in the experiment (Conboy and Kuhl, 2011; Conboy et al., 2015). Infants’ productive and receptive vocabulary development have also been linked to their ability to follow gaze and attend to the same referents as their communicative partners (Carpenter et al., 1998; Brooks and Meltzoff, 2008, 2015; Tenenbaum et al., 2015; Yu et al., 2019; Bruce et al., 2022). Attention to social stimuli and its relation to language development was further suggested in a study that measured infants’ gaze at 11 months of age to a social stimulus (i.e., a video of an adult female telling an engaging story using IDS) versus to a non-social distractor (moving patterns); the authors report that infants who showed sustained attention (infants’ looking time) to the social stimulus had larger vocabulary size and multi-word productions at 18 months of age (Salley et al., 2013).

In the current study we examined whether individual differences in infant HR—measured as a proxy for attention during live, natural, mother–infant interaction—is related to infants’ speech input at home at 3 months of age and their 30-month language development scores in a longitudinal assessment of the same children. To test our hypotheses, we capitalized on a large, existing dataset that included multiple behavioral assessments of infants from 3 months to 2.5 years of age. In that cohort, in addition to behavioral measures (Endevelt-Shapira et al., 2024), we collected infant HR data at 3 months of age during mother–infant interaction in the laboratory. These infant HR physiological (electrocardiogram, ECG) data have not been analyzed before, and are here being analyzed to test the hypotheses of the current study.

We tested two specific hypotheses. First, we hypothesized that infant exposure to IDS and engagement in CTs in their home at 3 months of age is associated with infant heart rate during mother–infant interaction in the laboratory. Because attention and orienting responses in infants are associated with heart rate deceleration (Graham and Clifton, 1966; Richards and Casey, 1991; Petrie Thomas et al., 2012), we reasoned that higher IDS exposure should be associated with lower infant HR during social–linguistic interactions. To ensure adequate control comparisons in this correlational longitudinal study, we examined two additional speech input variables in the same infants in the same sessions: (a) the mother using IDS in a group context (as opposed to 1:1) and (b) the mother using standard speech (as opposed to IDS) in a 1:1 context, expecting no significant correlations would be found between these two control variables and infant HR. Second, we hypothesized that infant HR during early social interaction would be significantly associated with children’s later language development, such that a lower heart rate at 3 months during mother–infant social interaction would be associated with higher vocabulary scores at 30 months.

## 2 Methods

### 2.1 Participants

Thirty-one mother–infant dyads (mothers’ mean age at the first visit was 32.9 ± 3.0 years, infants’ mean age at the first visit was 77.2 ± 6.7 days, 12 boys, 19 girls). The pre-established inclusion criteria included the following: (a) full term and born within 14 days of the due date, (b) no known health problems and no more than 3 ear infections, (c) birth weight ranging from 6 to 10 lb, (d) no significant foreign language exposure (i.e., parents and regular caregivers speak only English to the infant), and (e) all mothers were first-time parents. Socioeconomic status (SES) was assessed using the Hollingshead index (Hollingshead, 1975), based on parental education level and occupation (*M* = 54.3 ± 8.9, range = 19–66). All experimental procedures were approved by the University of Washington Institutional Review Board, and all participating families gave informed consent and were compensated monetarily for their time and effort.

### 2.2 Design and procedure

The experimental design included three widely used and validated tools, as summarized in Table 1. First, to characterize the physiological (HR) patterns of infants during mother–infant 1:1 social interaction, the mothers and the infants were invited to the laboratory and participated in a free play interaction session in the lab while electrocardiogram (ECG) was recorded. Second, within 1 week following this in-laboratory free-play social interaction session, the families completed LENA (Language ENvironment Analysis system, LENA) recordings in their home environment for the assessment of language input in natural settings over two consecutive days (infants’ mean age at the time of the LENA recording was 82.2 ± 8.9 days).

**Table 1:** Tools and variables used in the current study.

Tool	Variables	Variable definition
ECG (assessed at 3 months)	Infant heart rate (HR)	ECG was recorded from infants during 5 min of mother–infant face-to-face interaction
LENA (assessed at 3 months)	Mother infant-directed speech (IDS)-1:1	Mother spoke directly to the infant, IDS style was used, and only the mother’s voice was recorded during the interval
	Mother infant-directed speech (IDS)-group	Mother spoke directly to the infant, IDS was used, and two or more adult voices were recorded during the interval
	Mother standard speech-1:1	Mother spoke directly to the infant, standard (ordinary) speech was used, and only the mother’s voice was recorded during the interval
	Mother–infant conversational turns (CTs)-1:1	Mother’s utterances followed within 5 s by a child utterance, or vice versa, and only the mother’s voice was recorded during the interval
CDI (assessed at 30 months)	Vocabulary percentile scores	Parents check all words the child can produce out of 680 words
	Irregular words percentile score	Parents check all irregular words the child can produce out of 25 irregular nouns and verbs

Third, language development was assessed when the infant participants reached the age of 18 months and continued to 30 months of age using the MacArthur-Bates Communicative Development Inventory (CDI, Fenson et al., 1994). For the purpose of the current study, we use CDI scores of the latest time point collected, that is, 30 months of age.

#### 2.2.1 Electrocardiogram (ECG)

To characterize the physiological (HR) patterns we used ECG recordings at 3 months of age during mother-infant typical interactions. During the interaction the infants were sitting in an infant seat facing the mothers. The mothers were given the option to choose between sitting on the carpet or on a low couch facing the infant. The interactions were recorded using low-quality video recorders for the purpose of monitoring the session. ECG was recorded from infants during 5 min of mother–infant face-to-face interaction using a BIOPAC MP160 (BIOPAC Systems, Inc., Goleta, CA, United States) system at a sampling frequency of 2 KHz. Electrodes were applied in a three-lead, chest-mounted configuration with one electrode under each clavicle and the third on the lower left rib cage. Mothers were instructed to interact as they typically would at home with their infant for 5 min. Electrocardiogram (ECG) signals were sent to the MP160 with a BioNomadix wireless transmitter. QRS peaks were detected using AcqKnowledge data analysis software (Biopac systems). The data were visually inspected to detect artifacts, and manual corrections were performed when needed. Following this procedure, the peak list of each participant was analyzed using Python to calculate the Infant HR variable.

#### 2.2.2 Language ENvironment Analysis System, LENA

To assess everyday social interactions between adults and infants in natural settings we used LENA system (Language Environment Analysis Foundation, Boulder, Colorado). The LENA recorder can store up to 16 h of digitally recorded sound and can be snapped into a pocket on the front of a child’s vest, and subsequently downloaded and analyzed by the LENA software. The LENA software provides several automated measures of the language content, but for the purposes of the present study, all language measures were coded manually, following procedures described in previous studies (Ramírez-Esparza et al., 2014; Ferjan Ramírez et al., 2020).

The LENA audio files were processed using the LENA Advanced Data Extractor Tool (ADEX) to automatically identify segments for further manual analyses. Each participant’s recordings were segmented into 30-s intervals. To identify intervals with language activity for each of the 2 recording days, 50 intervals (per day) with the highest number of adult words heard by the child that were at least 3 min apart, were automatically selected. Thus, a total of 100 30-s coding intervals per participant were identified (2 days x 50 intervals each day). Coders listened to each 30-s interval and entered a “Yes” or a “No” for each of the following speech input variables: (a) Mother infant-directed speech (IDS)-1:1, (b) Mother IDS-group, (c) Mother standard speech-1:1, and (d) Mother–infant CTs-1:1. Definitions used for manual coding are provided in Table 1. The resulting matrix of Yes and No responses for each 30-s interval indicated that a specific category occurred or did not occur in that interval. Then the percentage of intervals coded for each category were calculated.

#### 2.2.3 MacArthur-Bates communicative development inventory (CDI)

The CDI is a widely used tool for assessing child productive language development based on parental reports. In the current study, we used CDI percentile scores assessed at 30 months of age. Percentile scores provide individual’s relative position in comparison to a large norming sample for boys and girls. Two specific measures of language development scores were assessed: (a) *Vocabulary percentile scores* and (b) *Irregular words percentile score* (CDI variable definitions are provided in Table 1).

### 2.3 Exclusion data

Thirty-one mother–infant dyads were fitted with ECG electrodes. Four participants had technical issues during data acquisition, e.g., electrodes disconnecting during recordings, and were excluded from further analysis. Thus, the remaining analyses included 27 infants with ECG data. In addition, each of the following analyses included a different number of subjects: one participant was excluded from maternal speech input and Infant HR analysis because their %Mother IDS-1:1 exceeded more than 2.5 standard deviations from the group mean (group mean following exclusion = 22.6 ± 10.7%, resulting in 26 participants for this analysis). In the analysis that included CDI scores, one dyad was not included in the analysis because the parents did not complete the CDI forms, and three additional participants were excluded because their vocabulary output scores at 30 months were lower than 2.5 standard deviations from the group mean (group mean following exclusion = 576.5 ± 103.1 words, resulting in 23 participants for this analysis).

### 2.4 Statistics

All statistical analyses were conducted using JASP (Version 0.18.2). Spearman correlations were used to assess the relation between Infant HR, speech input variables, and language development scores. Spearman partial correlations were also conducted to control for the effects of SES on the relation between Infant HR and speech input variables. Moreover, Spearman partial correlations were also conducted to control for the effects of speech input (%Mother IDS-1:1 and %Mother–infant CTs-1:1) on the relations between Infant HR and language development scores. A *p*-value less than 0.05 was considered statistically significant.

## 3 Results

### 3.1 Correlations between infant HR and speech input

We assessed whether Infant HR during face-to-face interaction with their mothers was related to speech input scores obtained from the LENA home recordings during mother–infant 1:1 interaction at 3 months of age. The speech input variables used in the analysis include: (a) %*Mother IDS-1:1*, (b) *%Mother IDS-group*, (c) *%Mother Standard speech-1:1*, and (d) *%Mother–infant CTs-1:1* (See Table 2 for descriptive statistics of the variables).

**Table 2:** Descriptive statistics of study variables.

Variable	*n*	Mean	*SD*	Median	Range
Infant HR (bpm)	27	152.4	7.6	151.5	133.1–165.4
%Mother IDS-1:1	26	22.6	10.7	21.5	4–43
%Mother IDS-group	26	17.5	7.5	18.0	4–31
%Mother Standard speech-1:1	26	10.2	7.5	9.0	0–22
%Mother–infant CTs-1:1	27	1.5	1.8	1.0	0–6
Vocabulary Percentile scores	23	65.2	27.9	75	14–98
Irregular words Percentile scores	23	52.8	35.9	43	7–99

**Figure 1:**
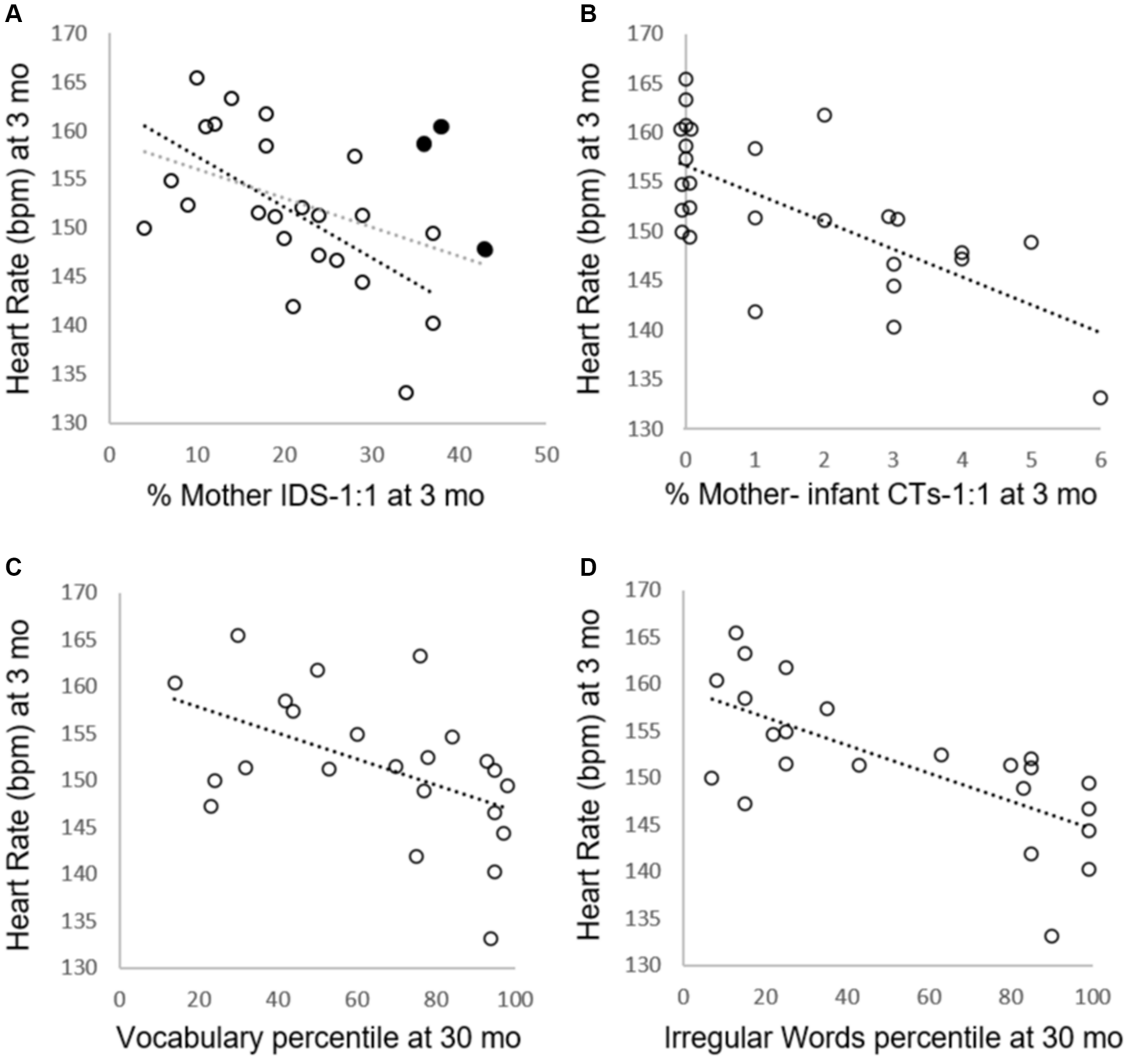
Infant heart rate and its relation to speech input and language outcomes. Scatter plots showing the associations between infant heart rate during mother–infant interaction at 3 months of age in the laboratory and each of four other measures. Each circle represents an infant. **(A)** Infant HR association with %Mother IDS-1:1 at 3 months of age. The gray dotted line is the correlation of all 26 subjects. The black dotted line is the correlation of 23 subjects, after excluding three outliers with low CDI language scores (shown in filled circles), see main text for details. **(B)** Infant HR association with %Mother–infant CTs-1:1 at 3 months of age. **(C)** Infant HR at 3 months of age association with Vocabulary percentile scores at 30 months of age. **(D)** Infant HR at 3 months association with Irregular words percentiles scores at 30 months of age.

We used Spearman correlations to assess the relations between Infant HR and speech input variables (Table 3). The analysis revealed that Infant HR at 3 months of age is negatively and significantly correlated with %Mother IDS*-*1:1 (*n* = 26, *r*_s_ = −0. 47, *p* = 0.015) (Figure 1A). As expected, no significant correlations were found between %Mother IDS-group and Infant HR (*n* = 26, *r*_s_ = −0.34, *p* = 0.09) or %Mother Standard-1:1 and Infant HR (*n* = 26, *r*_s_ = −0.20, *p* = 0.34). Moreover, when excluding three participants with outlier CDI vocabulary scores (lower than 2.5 standard deviations from the group mean), the strength of the correlation between %Mother IDS*-*1:1 and Infant HR increased (*n* = 23, *r*_s_ = −0.65, *p* < 0.001). A negative and significant correlation was observed between %Mother–infant CTs-1:1 and Infant HR (*n* = 27, *r*_s_ = −0.69, *p* < 0.001) (Figure 1B). At this early age, only 52% of the sample had %Mother–infant CTs-1:1 (14 out of 27 dyads). To further check the relation between Infant HR and %Mother–infant CTs-1:1, we split the %Mother–infant CTs-1:1 scores into two subgroups using a median split (Median = 1), resulting in a group of 13 dyads with a score of 0 (no Mother–infant CTs-1:1) and 14 dyads with at least 1 CT (Range = 1–6). We then compared Infant HR between the two subgroups using the non-parametric Mann–Whitney test. The analysis revealed significant differences in Infant HR between the groups (*U* = 26, *p* = 0.001), and as expected, there was significantly lower HR among the infants with CTs (mean HR = 148.2, *SD* = 7.2) compared to those without CTs (mean HR = 156.9, *SD* = 5.1).

**Table 3:** Correlations between speech input variables (LENA) and Infant HR.

Speech input variable	Infant HR
%Mother IDS-1:1	*r* = −0.47 *p* = 0.02^*^
%Mother IDS-group	*r* = −0.34 *p* = 0.09
%Mother Standard-1:1	*r* = −0.20 *p = 0.34*
%Mother–infant CTs-1:1	*r* = −0.69 *p* < 0.001^***^

^*^p < 0.05, ^***^p < 0.001. *r*, Spearman’s correlation coefficient; *p*, *p*-value.

Previous studies indicate that socioeconomic status (SES) is a factor associated with language development (Hart and Risley, 1995; Fernald et al., 2013) and with IDS in the context of 1:1 interaction (Ramírez-Esparza et al., 2014). In the current study we found that SES (*N* = 27, mean = 53.9, *SD* = 9.4, median = 54.5, range = 19–66) was negatively correlated with %Mother IDS-1:1 (*r*_s_ = −0.55, *p* = 0.003) but not correlated with language development scores (both |*r*_s_| < 0.32, both *p* > 0.15). To further check these results, we conducted partial spearman correlations between Infant HR and speech input variables controlling for SES (see Supplementary Table S1) and we obtained the same pattern of results.

### 3.2 Correlations between infant HR at 3 months of age and productive language development at 30 months of age

We also tested whether Infant HR during face-to-face interaction with their mothers at 3 months of age was related to language development at 30 months of age, exploring both *Vocabulary percentile scores* and *Irregular words percentile scores* (see Table 2 for descriptive statistics). The analysis revealed that Infant HR at 3 months of age is negatively and significantly correlated with Vocabulary percentile scores at 30 months of age (*n* = 23, *r*_s_ = −0.51, *p* = 0.014) (Figure 1C) and with Irregular words percentile scores at 30 months (*n* = 23, *r*_s_ = −0.70, *p* < 0.001) (Figure 1D).

Because the two speech input variables, %Mother IDS-1:1 and %Mother–infant CTs-1:1, are significantly correlated with both Infant HR (Table 3) and language development scores (Table 4), we used partial Spearman correlations to account for their effect on the correlations between Infant HR and language development scores. The analysis revealed a significant correlation between Infant HR at 3 months of age and Irregular words percentile scores at 30 months of age (*r*_s_ = −0.48, *p* = 0.034), but not with Vocabulary percentile scores (*r*_s_ = −0.34, *p* = 0.14).

**Table 4:** Correlations between measurements assessed at 3 months of age (Infant HR and speech input variables) and language development scores at 30 months of age.

Variable	Vocabulary Percentile scores	Irregular words Percentile scores
Infant HR	*r* = −0.51 *p* = 0.014^*^	*r* = −0.70 *p* < 0.001^***^
%Mother IDS-1:1	*r* = 0.51 *p* = 0.015^*^	*r* = 0.76 *p* < 0.001^***^
%Mother–infant CTs-1:1	*r* = 0.22 *p* = 0.32	*r* = 0.43 *p* = 0.04^*^

^*^p < 0.05, ^***^p < 0.01.

### 3.3 Correlations between infant HRV, speech input and language development

In an exploratory, supplementary analysis (helpfully suggested by a reviewer) we also looked at the relation between infant heart rate variability (HRV) to speech input and language development. We found that Infant HRV is positively and significantly correlated with %Mother–Infant CTs-1:1 (*r* = 0.43, *p* = 0.03) and with Irregular words percentile scores (*r* = 0.43, *p* = 0.04). However, unlike Infant HR, Infant HRV was not significantly correlated with %Mother IDS-1:1 (*r* = 0.20, *p* = 0.32) or Vocabulary percentile scores (*r* = 0.34, *p* = 0.11) (Supplementary, Table S2).

## 4 Discussion

The goal of the current study was to examine the relations between infant heart rate as an index of attention during one-on-one (1:1) parent–infant interaction at 3 months of age and measures of speech input at 3 months of age, as well as with language outcomes in these same children at 30 months of age in a longitudinal study. Several novel findings emerged.

We found that %Maternal IDS-1:1 is negatively and significantly correlated with the physiological measure of infant heart rate. More specifically, a higher occurrence of maternal IDS in the home environment during 1:1 interaction is associated with lower infant heart rate measured during mother–infant interaction in the laboratory. This finding is in line with previous studies that explored the relation between infant heart rate and exposure to social stimuli such as speech input, which found a decrease in infant heart rate during exposure to IDS (Santesso et al., 2007; Curtindale et al., 2019; Lin et al., 2023). The authors speculated that IDS is attentionally engaging (Santesso et al., 2007). In addition, a more recent study investigated infants’ attention to a social stimulus using both look duration and infant heart rate. The social stimulus was a video of an adult female reciting three phrases with positive affect in a continuous loop of IDS, and the non-social stimulus was a red hammer moving up and down, tapping on a wooden surface. The authors found that infants’ look durations were longer and that infants’ heart rate was significantly lower during the social stimulus versus non-social stimulus (Curtindale et al., 2019). The relation between IDS and slower heart rate is further supported by a study that exposed high-risk newborns to recordings of their parents’ voices. The stimuli included a recording of their mother or their father reading a children’s book. Results showed that higher-pitched voices, which is one of the main characteristics of IDS, were correlated with slower heart rates (Lin et al., 2023).

The foregoing pattern of results, taken together with long-established findings that infants’ attention and orienting responses are associated with heart rate deceleration (Graham and Clifton, 1966; Petrie Thomas et al., 2012), and our current findings showing a lower heart rate for infants who hear more IDS at home (LENA records), suggests that experience with IDS may tune infants’ attention to speech. From birth, infants prefer listening to the IDS speech register (Fernald, 1985; Cooper and Aslin, 1990), and it has been argued that IDS modulates infants’ attention and arousal in a way that supports real-time communication and learning. More recently, it was proposed that IDS supports speech processing by optimizing neural entrainment, where neural oscillations become time-locked to key moments in an attended stimulus, thus enhancing time-locked attention (Goswami, 2019; Nencheva and Lew-Williams, 2022).

The two additional control analyses reported here provide useful corroborating information. In one, we measured mothers’ standard speech in a 1:1 context and did not find a significant correlation between this speech variable and infant heart rate, suggesting that the *quality* of the speech input is a critical factor in the observed correlation between speech input at home and infant heart rate. In a second control, we measured the relation between the mothers’ infant-directed speech in a group setting and infant heart rate. The analysis also revealed no significant correlation, suggesting that (in addition to speech quality) the 1:1 adult–infant *context* is a relevant factor. Taken together, this pattern of results suggests the potency of infant-directed speech in the context of 1:1 social interaction in tuning infants’ attention to language. We speculate that during 1:1 interactions, in contrast to interactions in group settings, infants experience more contingent social reactions, and parents maintain direct gaze with the infant which may further facilitate infants’ attention to speech.

Going beyond behavioral and physiological measures, there are some relevant brain studies that also bear on the current findings. The importance of the combination of infant-directed speech and direct gaze was also noted in a functional near-infrared spectroscopy (fNIRS) study that used a naturalistic interaction design. In that study, 6-month-old infants’ cortical responses to IDS were enhanced when speech was presented with direct eye contact (Lloyd-Fox et al., 2015). This effect was indicated in brain regions known to be involved in processing auditory and visual aspects of social communication. Moreover, a recent study (Bánki et al., 2024) assessed how communicative signals such as eye contact, infant-directed speech, and pointing simultaneously affect the brain activity of 11–12-month-old infants and their caregivers engaging in a naturalistically designed joint attention situation. Infants showed increased visual processing of objects during joint attention that occurred with versus without communicative cues, as indexed by greater neural responses at central, parietal, and occipital EEG electrode sites during joint attention to the presented images. The authors interpreted these results as indicating the role of communicative cues in information processing during social learning in infancy.

In addition to speech style and context we found that mother–infant conversational turns are negatively and significantly associated with infant heart rate, such that, higher use of conversational turns is associated with lower infant heart rate. This finding emphasizes the importance of bidirectional communication and suggest its role in increasing infants’ involvement and attention to the interaction, which is indicated by lower heart rates. Moreover, the analysis with Infant HRV revealed that among the LENA variables used in this study, only mother–infant CTs were significantly and positively correlated with Infant HRV (Supplementary Table S2). This finding makes sense in that it is consistent with previous studies showing a link between higher HRV and better performance in cognitive tasks, including attention, executive function, memory, and linguistic performance [see Forte et al. (2019) and Thayer et al. (2009) for review]. While the other speech input measurements do not take into account infant’s engagement or performance, CTs reflect an active engagement of the infant during social interaction.

Finally, we found strong prospective negative correlations between infant heart rate at 3 months of age and later language development at 30 months of age, such that lower heart rate during mother–infant interaction was associated with higher language development (CDI) scores. These results are in line with a recent study that explored the link between attention during social play at 3–4 months and early prelinguistic social–communication skills at 9 months of age. In this study, the authors report that greater heart rate deceleration during episodes of sustained attention was associated with later social–communication skills (Bradshaw and Abney, 2021).

We also find it intriguing that the correlation between infant heart rate at 3 months of age and language development scores at 30 months of age was stronger with irregular words percentile scores, and was significant also when accounting for the effects of speech input variables mothers’ infant-directed speech and conversational turns using partial correlations. Children’s early usage of correct irregular word forms is one of the first signs that they are learning the morphology of their language. This section of the CDI inventory asks parents to specify whether the child has begun to use each of five common irregular plural nouns and 20 common irregular past-tense verbs. Although some children produce irregular forms in their second year, it is in the third year that acquisition of irregular morphology accelerates (Fenson et al., 1994). The current findings suggest that attention to speech from early infancy is strongly associated with more complex aspects of language learning that are acquired later in development. Similarly, infant HRV was positively and significantly correlated with irregular words percentile scores but not with vocabulary percentile scores.

### 4.1 Conclusion, limitations, and future directions

Our results converge to support the hypothesis that a mechanism by which infant–caregiver interactions and speech—particularly IDS in 1:1 context—support language development is increased infants’ attention to speech (see also Bosseler et al., 2024). Attention in the current study was indexed by measures of infant heart rate. We show that higher IDS in 1:1 context is associated with lower heart rate in early infancy, and that both higher IDS and lower heart rate are significantly associated with infants’ future language development more than 2 years later. Our interpretation of the data is consistent with that of Masek et al. (2021), who argue that infant attention and contingent interaction reciprocally build on each other and foster the development of language and communication skills. Their model considers attention to be the glue that both enables infants to engage in contingent social interactions and then enables the contingent input to support learning (Masek et al., 2021).

In the current study it is noteworthy that infant heart rate and speech input were measured at a very early time window in development, 3 months of age, before the “sensitive period” for language learning. Infants are exposed to social information from birth and by 3 months of age, and rapidly begin to attune to the social environment, for example, by engaging in reciprocal exchanges during face-to-face social interactions with their caregivers (Lavelli and Fogel, 2002), participating in conversation-like turn-taking (Gratier et al., 2015), and changing their visual, facial, and vocal behaviors in response to their caregiver’s social cues (Meltzoff and Moore, 1998; Feldman, 2007; Beebe et al., 2016). We suggest that increased infants’ attention to social interaction and speech input during this early developmental window supports infants’ social brain growth and language learning.

Several study limitations of this study should be acknowledged. First, in the current study, we assume that speech input patterns, as recorded in the home environment, are similar to the interaction at the laboratory during ECG recordings. Future research should explore heart rate changes in response to concurrent language input during live social interaction. That being said, we speculate that infants who experience more mother–infant one-on-one interactions using IDS in their natural home environment are more attentive during interactions with their mothers. Therefore, even if the mother’s behavior and responses in the laboratory environment are not entirely natural and do not perfectly reflect what occurs at home, infants who experience more IDS at home are likely to be more attentive to the interaction in the laboratory and thus, their heart rate may be lower compared to infants who experience less infant-directed speech from mothers at home. Second, heart rate was only recorded during mother–infant interaction. There is no other condition for comparison, such as resting state or interaction with a strange person. Therefore, based on the current study, we cannot infer that the observed correlation is limited to Infant HR during moments of mother–infant interaction alone, because it could potentially be related to the differences in their baseline heart rates. Future research should employ a paradigm that includes a baseline condition with no social interaction or interaction with another adult (in addition to the mother). Third, we note that our findings represent a fairly small sample size, which was demanded by the rigors of longitudinal studies, and it will be important for future research to follow a larger sample to test whether the observed patterns are replicated and can be generalized to a broader population. In addition, an unexpected finding emerged, revealing a negative correlation between SES and IDS. We speculate that this correlation may be linked to the specific characteristics of the cohort participating in the current study (relatively high SES scores). Future research should include families with a much wider and more representative SES range. Finally, due to the continuous ecological nature of our paradigm, which requested that mothers interact in their own typical way with their infant, we are limited in our ability to investigate heart rate changes as a function of attention phases such as pre-attention, stimulus orientation, sustained attention, and attention termination.

To conclude, our current results support the hypothesis that infant learning in the context of “live” interactive social exposure may be at least partially accounted for by the increased attention and motivation produced by social interaction (Kuhl, 2007). The current study further emphasizes the importance of speech style (IDS) and social context (1:1 interactive context) in increasing infants’ attention to the social interaction, thus supporting language development. We believe that infant heart rate during social–linguistic interactions could be used as a useful physiological marker or predictor of children’s future language growth. Such physiological markers or predictors can serve to broaden current theories and empirical tools for advancing our understanding of the foundations and support for language acquisition.

## Data availability statement

The raw data supporting the conclusions of this article will be made available by the authors, without undue reservation.

## Ethics statement

The studies involving humans were approved by University of Washington Institutional Review Board. The studies were conducted in accordance with the local legislation and institutional requirements. Written informed consent for participation in this study was provided by the participants’ legal guardians/next of kin.

## Author contributions

YE-S: Conceptualization, Formal analysis, Writing – original draft, Writing – review & editing. AB: Conceptualization, Investigation, Methodology, Writing – review & editing. TZ: Methodology, Writing – review & editing. JM: Conceptualization, Project administration, Writing – review & editing. AM: Funding acquisition, Writing – review & editing. PK: Funding acquisition, Supervision, Writing – review & editing.
