# Supplementary material for: Heart-to-heart: infant heart rate at 3 months is linked to infant-directed speech, mother–infant interaction, and later language outcomes
Source: Front Hum Neurosci. 2024 May 2;18:1380075. doi: 10.3389/fnhum.2024.1380075 (PMC11096508; doi:10.3389/fnhum.2024.1380075)
Supplement: Supplementary file 1 [file Table_1.DOCX]

Supplementary Material

**Heart-to-heart: Infant heart rate at 3 months is linked to infant-directed speech, mother–infant interaction, and later language outcomes**

**Yaara Endevelt-Shapira^1*^, Alexis N. Bosseler^1^, T. Christina Zhao^1,3^, Julia C. Mizrahi^1^, Andrew N. Meltzoff^1,2^, Patricia K. Kuhl^1,3^**

^1^Institute for Learning & Brain Sciences, University of Washington, USA

^2^Department of Psychology, University of Washington, Seattle, Washington, USA

^3^Department of Speech and Hearing Sciences, University of Washington, USA

*** Correspondence:**

Yaara Endevelt-Shapira
[yaaras@uw.edu](mailto:yaaras@uw.edu), 425-539-8811

**Table S1**

*Correlations between infant heart rate and speech input variables (LENA) controlling for SES*

| Speech input Variable | Infant HR |
| --- | --- |
| %Mother IDS-1:1 | *r = -0.44*  *p* = 0.03* |
| % Mother IDS-group | *r = -0.29*  *p* = 0.16 |
| %Mother Standard-1:1 | *r = -0.14*  *p = 0.50* |
| %Mother–Infant CTs-1:1 | *r = -0.67*  *p* < 0.001***** |

*Note: * p < .05, *** p < .001*

**Table S2.**

*Correlations between Infant HRV (SDNN,* the standard deviation of all R–R intervals*) and speech input variables (LENA) and language development scores (CDI)*

| Speech input Variable | Infant HRV |
| --- | --- |
| %Mother IDS-1:1 | *r = 0.20*  *p* = 0.32 |
| % Mother IDS-group | *r = -0.17*  *p* = 0.41 |
| %Mother Standard-1:1 | *r = -0.06*  *p = 0.79* |
| %Mother–Infant CTs-1:1 | *r = 0.43*  *p* = 0.03*** |
| Vocabulary  Percentile scores | *r = 0.34*  *p* = 0.11 |
| Irregular words  Percentile scores | *r = 0.43*  *p* = 0.04*** |

*Note: * p < .05*
